# Power through: A new concept in the empowerment discourse

**DOI:** 10.1016/j.gfs.2019.07.001

**Published:** 2019-06

**Authors:** A. Galiè, C.R. Farnworth

**Affiliations:** a International Livestock Research Institute, ilri.org Box 30709, Nairobi, Kenya; b Pandia Consulting, Researcher on gender, agricultural value chains, climate change, Germany

## Abstract

• Current empowerment efforts fail to capture a critical component of empowerment - its relational nature.
• Individual's empowerment is constituted through the empowerment of others in significant relationship to them.
• The ability of women to empower themselves is mediated through the deliberative support of others.
• Community judgment of individual alignment with gender norms determines empowerment outcomes in daily realities.
• Public adherence to local gender norms creates a safe façade to deviate from the norms.

## 1 Introduction

Women's empowerment describes the capability of women for self-determination: to take control over their own circumstances and to realize their aspirations in order to live a life they have reason to value (Annas, 2003; Kabeer, 1999; Sen, 1990). The emphasis is on ‘agency’ described by Kabeer (1999) as the ability to define goals, have meaningful choices, and to act to achieve desired outcomes (see also Ibrahim and Alkire, 2007; Nussbaum, 1999; Sen, 1999). Women's agency can be exercised at the individual cognitive level (e.g. reflection and analysis), as well as at relational and collective societal levels (e.g. decision-making, negotiation, manipulation, resistance) (Cathy Rozel Farnworth et al., 2018a, Farnworth et al., 2018b; Yount et al., 2015).

Many understandings of empowerment are individualistic because they focus on facilitating the expansion of an individual's capabilities to achieve empowerment on her own, and on her own terms (Bhattarai and Pant, 2013; Fernea, 2003; Ibrahim and Alkire, 2007; Nussbaum and Sen, 1993; Riger, 2002). However, women's empowerment can equally be understood as a multi-dimensional process that perforce entails social relations among individuals, groups of people, and institutions (Acharya et al., 2010; Bayissa et al., 2018; Belcher et al., 2011; Doss, 2013; Kabeer, 2011; Mokomane, 2012). This is because women's empowerment is contingent not only upon changes within individual persons, but also on the ways power structures relationships within and between different institutional levels (Nazneen et al., 2014; Sen, 1994). Eger et al. (2018) explain that empowerment processes are inevitably affected by social norms and discourse and therefore involve relational, multi-level and multi-directional processes of change. In many societies, also, individual empowerment is associated to a strong sense of family togetherness (Mokomane, 2012). Women need to be attuned to the demands of the people with whom they live when they seek to negotiate their way towards personal empowerment (Belcher et al., 2011; C. R. Farnworth et al., 2018a, Farnworth et al., 2018b; Mokomane, 2012). Kandiyoti, (1988) formulates the ‘patriarchal bargain’ by explaining that women strategize within a set of specific constraints that call for different strategies to maximize security and optimize life options with varying potential for active or passive resistance in the face of oppression. Such strategizing assumes a high level of consciousness and forward-planning (Farnworth et al., n.d.).

Social norms clearly play a role in determining ‘the possible’ when it comes to the choices women may decide to make and to enact. Social norms lie outside the immediate control (or agency) of individuals and can greatly influence individual choice. Stewart, 2013 argues that no one can experience complete autonomy: alongside political and economic constraints, their choices are heavily influenced by underlying social norms (Farnworth et al., 2017, n.d.). The theory of doxa put forward by Bourdieu (1977: 167) suggests that some social norms are so deeply embedded ‘in our ways of thinking and acting that we […] follow them unconsciously and without deliberation’. Gender norms, in this understanding, are the socially constructed and accepted roles and stereotypes ascribed to gender that are naturalized in people's behaviour: a kind of unquestioned truth which people live by (Farnworth et al, nd).

### 1.1 From power to empowerment: four definitions

To get a better grasp on the concept of ‘power’ within the word ‘empowerment’ four definitions of power have been developed over time and are now widely used. They attempt to capture both what the terms empowerment and disempowerment actually constitute, and how they are enacted within, and between, individuals (Munoz Boudet et al., 2012). ‘**Power within**’ refers to a transformation of individual consciousness which leads to a new self-confidence to act (Rowlands, 1997). ‘**Power with**’ is power that results from individuals organizing and acting as a group to address common concerns (Gammage et al., 2016). Cornwall (2016) terms this the ‘sociality’ or ‘solidarity’ that the processes of collective empowerment entail. ‘**Power to**’ is the power to bring about an outcome or resist change. Allen, (1999) terms this the ‘power to act’ — often associated with ‘empowerment’. ‘**Power over**’ suggests a social relation of domination or subordination between individuals (Pansardi, 2012).

These four definitions of empowerment seem to share a conceptualization of power which becomes visible through the enactment of ‘agency’ in different ways. Power also appears to be something like a ‘property’ which can be ‘owned’ by an individual (‘I am empowered’). (Although ‘power with’ describes the co-creation of power by groups of individuals it nevertheless conceptualizes of power as a property residing in, and confined to, individuals who act collaboratively to increase their individual power through group action.)

**Fig. 1:**
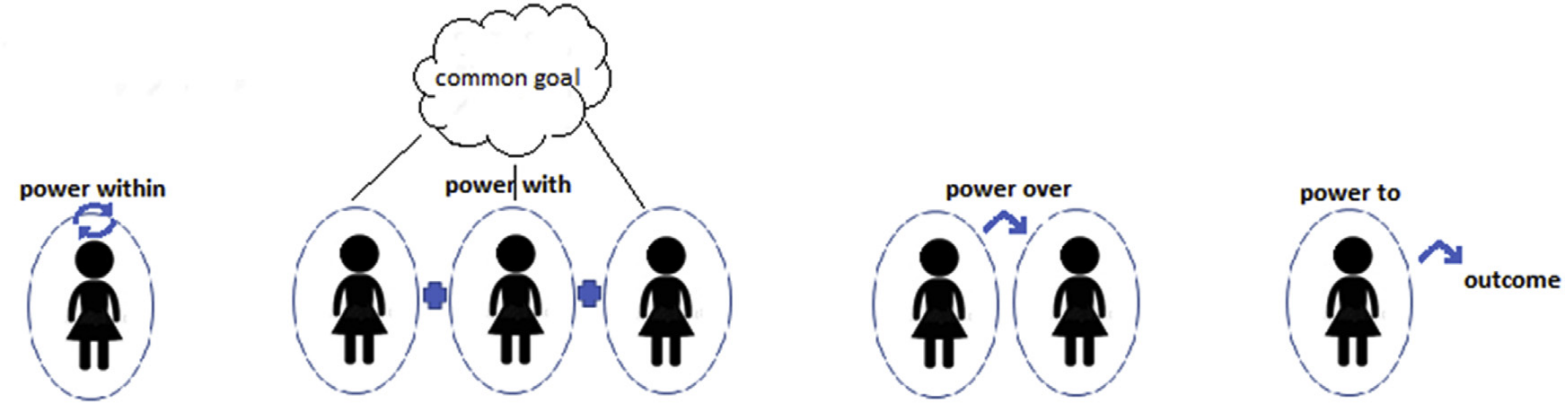
Representation of the four definitions of power.

Source: authors' elaboration

Fig. 1 is a visual representation of these four different definitions of power. Each definition implies that there is an empowerment boundary which is intrinsically associated with individuals. At the same time the definitions fundamentally recognize that an individual empowerment boundary is capable of expanding to accommodate an increase in personal empowerment. In Fig. 1, the oval around each individual represents their empowerment boundary. In the case of ‘power with’ individuals come together to act intentionally towards a common goal, the aim of which is to expand the boundary of empowerment of each person which can only be achieved through the empowerment of the whole.

### 1.2 Moving towards a new definition of power

The authors of this Perspective conducted empirical studies in three countries to try and develop an understanding of how people themselves conceptualize, and experience empowerment. We asked interview participants to describe their experiences of moving from disempowerment to empowerment, and back again in some cases. The findings suggest that the four definitions of empowerment, despite their richness and range, fail to capture something important in the way empowerment can be experienced. The concept of ‘*power through*’, proposed by the lead author and equally co-developed with the second author, aims to define this analytically distinct dimension of power. Here, we outline key features of this new concept and then elaborate upon them through reflecting on fieldwork findings.

At its broadest, the concept of *power through* captures an involuntary aspect of empowerment and disempowerment. As noted above, it is well understood that power can have normative dimensions which allow it to exist and be exercised in the absence of any apparent agency. However, *power through* adds a new dimension to power without agency: that of individual power won, and lost, through changes in the empowerment status of others, or through relating to others. In this process, the individual may not have acted. Rather, changes in the empowerment of individuals are mediated by 1. the empowerment status of significant people associated with them - parents, siblings, spouses, children, other relatives; 2. The way personal characteristics are considered to affect how an individual relates to others and; 3. the judgment by the immediate community within which they live. Through this process, the concept of *power through* allows the experience of empowerment and disempowerment to remain distinctive and personal to an individual, yet, mediated through the existence of others.

**Fig. 2:**
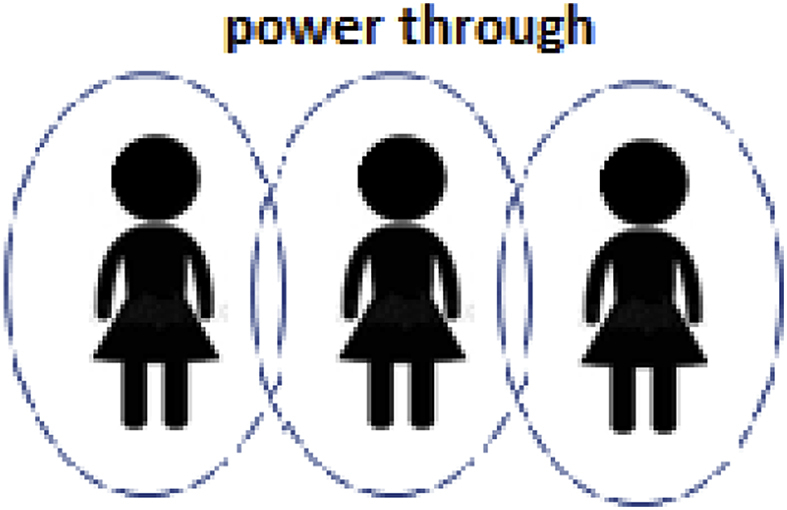
Representation of ‘power through’ introduced in this Perspective.

Source: authors' elaboration

Fig. 2 is a visual representation of ‘power through’. Here, the boundaries of individual empowerment overlap with those of others. The empowerment of one individual may change even if he or she does not act but rather because the empowerment status of significant others changes. Or, it may change because significant others allow or deny an individual the opportunity of empowerment.

This Perspective explores the empirical evidence that prompted the development of the concept of *power through* and discusses its conceptual and methodological implications. Although the concept is based on a limited set of findings the authors aim to stimulate further discussions on an aspect of empowerment – that of its relational and non-agentic nature - that needs further scrutiny.

## 2 Research methodology

This Perspective builds on qualitative fieldwork conducted between 2006 and 2017 in agricultural communities in Syria, Kenya and Tanzania. The fieldwork consisted of individual semi-structured interviews, and single-sex focus group discussions (FGDs) to explore local understandings of empowerment and the way empowerment-related experiences are lived by women and men. The main research question was: ‘what does empowerment mean to you?’ In cases where no clear concept of empowerment existed, we explored individual life aspirations over a 10-year time period and discussed under what circumstances each individual could achieve such aspirations. We then explored at length the way participants experienced instances of empowerment or disempowerment. In Syria, the study was conducted between 2006 and 2011 with 12 women and 24 men crop farmers from three villages - Ajaz, Souran and Lahetha. The participants were involved in a participatory plant breeding program coordinated by the International Centre for Agricultural Research in the Dry Areas (ICARDA) (Galiè et al., 2017). In Tanzania the fieldwork was conducted in 2017 in four districts, Kilosa, Handeni, Lushoto and Mvomero, with 24 men and 24 women dairy livestock keepers as part of a dairy intensification project, ‘MoreMilkin Tanzania’, coordinated by the International Livestock Research Institute (ILRI) (Galiè et al., 2018). Fieldwork in Kenyawas undertaken in 2017 and comprised FGDs with 20 men and 22 women milk traders from peri-urban Nairobi involved in the MoreMilk project, coordinated by ILRI, which assists milk traders to improve milk safety (Galiè et al., n.d.).

## 3 Findings and discussion

### 3.1 *Power through* by association with significant others

Syrian women participants were clear that their individual empowerment was largely determined by their association to others. If a family member obtained a prestigious or influential job, all his or her family members would automatically experience empowerment because the characteristics associated with that job would be ‘transferred’ by their community to the job holder and their family members. Having a higher social status was considered to improve the ‘potential’ scope of their future personal agency and therefore empowerment. That is to say, women felt that an increase in their social status through that of others enhanced their own ‘involuntary empowerment’ because it created the preconditions for the enactment of an agentic form of future personal empowerment.

The participants added that their personal empowerment is affected by assets owned by male family members. These can confer status upon them even though they do not personally own them. Indeed, when asked about their ownership of productive assets, women crop farmers - particularly young women, were unable to distinguish the assets that they owned from those owned by male family members. They called them ‘family assets’ even though they were registered under the men's names. Their inability, or unwillingness (?) to distinguish own versus family-owned assets was not related to ignorance regarding whose name an asset was officially registered under, nor to different understandings of what ownership could entail (Galiè et al., 2015), nor to a display of modesty. Rather, women felt that the overall wealth of the woman's family increased the perception others had of the personal wealth of the women, even though they did not actually own any assets personally, or, in the case of young women, expect to take any of these assets with them upon marriage. The women explained that being considered wealthy offered them more options to be empowered in the future by, for example, marrying into a wealthy family, or having more decision-making in the community.

The Syrian case study further indicated that the disempowerment of an individual can become collective by being ‘transferred’ to significant people associated to them. In 2010, for example, a young woman gave a presentation at an international conference in Aleppo without being chaperoned by an older family member. She translated her newly gained independence and self-confidence in a higher empowerment self-assessment quantitative score as compared to the score she gave herself prior to the conference (Galiè et al., 2017). Yet upon her return home, her wider community condemned her conduct and indeed the moral conduct of her whole family for allowing her to travel unsupervised. The young woman's disempowerment was transferred to her family with the result that community members stopped paying visits and the whole family - including male members - was ostracised.

For the Syrian participants, then, the processes of individual empowerment and disempowerment seem to lie, to a degree, beyond personal control. These findings have implications for how empowerment is conceptualized. They show that the empowerment of individual women is partly constituted through the empowerment of significant people - particularly men - associated with them. When a man in the family becomes empowered in the eyes of the community then the whole family - women and men alike - are considered empowered simply through association with that person.

### 3.2 *Power through* intrinsic personal characteristics and relating to others

Discussions with Kenyan men and women dairy farmer participants allow a different nuance to *power through* to emerge. They argued that an individual's empowerment is mediated through intrinsic (inborn) characteristics, with particular importance given to how these characteristics promote the ability to relate to others.

When exploring the characteristics of an ‘empowered man’ and an ‘empowered woman’, participants listed characteristics such as good health, self-confidence, skills, determination, and plenty of energy as necessary for both genders. Yet, they argued, these same characteristics have the potential to ‘interfere’ with an individual's ability to positively relate to someone of the other gender. If misapplied, they thought, these characteristics can become an active impediment to empowerment. For instance, the intrinsic characteristics of determination and self-confidence were argued to be necessary for women and for men to become empowered. However, whilst men expressing these characteristics are widely recognized to be empowered, participants were concerned that women with the same characteristics could disrespect their husband. A non-respectful wife is not acknowledged as empowered by the rest of the community.

In this case an involuntary form of empowerment emerges: a complex interplay between a woman holding innate characteristics of empowerment and the compatibility of these characteristics with locally sanctioned gender roles. This interplay creates the preconditions based on which women may decide to exercise their agency (i.e., decide how to utilize their ‘innate characteristics of empowerment’ when relating to others, in order to abide by local gender norms and ultimately, be considered empowered by their community).

Similarly, Tanzanian men and women participants explained that women could be empowered ‘even though’, or possibly ‘only when’, they have no final decision-making in households because having final decision-making was considered incompatible, for women, with their role as a ‘good wife’. By way of contrast, men's empowerment depends less on their successful enactment of their role as ‘good husbands’, and more on their relations with others in the community. A self-confident man, for the participants from Tanzania, was empowered because he supported the community by showing leadership skills and financial support for others.

The findings show that interplay between personal characteristics of empowerment and relating to others affect empowerment outcomes for both women and men but through different pathways. This is not as simple as saying that empowered men support the community and empowered women support their husbands: the degree of self-realisation differs - ‘empowered’ women clearly have less scope for realising their capacities. Women find themselves in a double bind - possessing characteristics considered to be innate which could assist them towards empowerment and, at the same time, being disempowered by these same characteristics which the community considered possibly incompatible with ‘relating to others according to appropriate gender roles’.

### 3.3 Power through community judgment and the gender norms façade

Five women participants from Tanzania explained how a process they initially experienced as empowerment rapidly translated into disempowerment because they had undermined gender norms. Their engagement in the market, as a result of a dairy intensification project, increased their income and consequently decreased their reliance on their husbands' intermittent contribution to family expenses - ‘a step towards empowerment’ in their opinion. However, due to this very independence their husbands reduced their contribution to household expenses, and in three cases, left the family. The women became main/sole providers of income and food to their family with the consequence that their vulnerability to food insecurity increased. The women's attempts to increase income, in the eyes of the community and their husbands, visibly undermined men's roles as breadwinners and their role as ‘dependent wives’. The men had felt disempowered and left; women felt now more disempowered than before. These findings demonstrate how undermining the personal status associated with men's gender roles as ‘provider’ outweighed, for three men, the benefits of women's strengthened financial contribution to household income.

In this case, as well as in the other case studies presented, the findings show that assessment by community members of the alignment between an individual's ‘gender performances of social roles’ and locally valid gender norms ultimately determines whether an individual is accorded an ‘empowered’ or ‘disempowered’ status. These perceptions in turn affected an individual's ability to enact their individual potential for empowerment. At the same time, ‘reality’ intervenes. In many communities, the challenges of securing livelihoods (also affected by wider national and global change processes, and discourses around gender (Farnworth et al. forthcoming, 2019)) are such that it is impossible for strict gender roles to be maintained in actuality. However, it was essential for households to be *seen* and *acknowledged* as conforming: all participants strived during our discussions to show that their whole family abided by locally valid gender norms. It was only after intensive interactions that participants began to acknowledge cases of non-compliance to these norms. We term ‘the gender norm façade’ this stereotypical descriptions by interview participants of their own household gender dynamics as reflecting the local gender norms, regardless of the actual performance of gender roles and dynamics (see also Farnworth et al., 2017, n.d.; Galiè, 2013; Rao, 2012).

The gender norms façade allows the necessity of change to be accommodated whilst at the same time avoiding open challenge to gender norms. The gender norms façade seems to create a safe space that permits rebellious (i.e. different from the norms) behaviours. Everyone in the community appears to ‘collude’ in the gender norms façade because doing things differently cannot go unremarked (Ball Cooper and Fletcher, n.d. on injunctive norms and sanctioning; see also Cislaghi and Heise, 2017). Over time, of course, society - and gender roles can change swiftly or incrementally (Farnworth et al., n.d.; Risseeuw, 2005).

## 4 Conclusion

The findings show that a person's ability to exercise agency, or not, rests to an important extent on processes beyond their personal control – a concept that we name *empowerment through*. Indeed, it appears that agency exists or is denied through processes that have an involuntary and sometimes barely articulated dimension. These processes create the preconditions based on which women can consciously decide to act to improve their empowerment status or otherwise.

The implications of the findings presented in this Perspective suggests that interventions aiming to empower women should carefully consider the interdependency and relatedness of empowerment. Specifically:

1. The empowerment of individuals is partly constituted through the empowerment of others in significant relationship to them (parents, siblings, spouses, in-laws, older children, etc.). This suggests that empowerment interventions must become a shared project of people significant to one another.
2. The concept of *power through* implies that research methodologies that attempt to measure the relative empowerment of individual women vis-à-vis individual men in a household have limited validity. Researchers may, for example, study individual empowerment as affected also by the community's view of this individual's household empowerment.
3. Interventions need to be designed with reference to locally valid cultural norms and how the process of *power through* is actually enacted in a particular community. The wider community, particularly important opinion formers, must be involved in developing and supporting empowerment initiatives. However, reference to cultural norms must not imply intention to reproduce them. Rather, it suggests a willingness to work with the community to identify and work with pride on strengthening the best, and to secure consent to change norms which are harmful.
4. The ‘gender norms façade’ allows men and women to deviate from gender norms without risk of sanction. It is important to understand the purpose of the ‘gender norms façade’ rather than simply attempting to expose it. Utilizing its strategic role means identifying sharper and sensitive strategies to leverage local gender norms that contribute to women's empowerment and which also empower men, for instance by assisting them towards a greater range of roles and self-expression.
5. The danger of the concept of *power through* is that it can be used to legitimize and reinforce male dominance. Measures to strengthen men could be considered a mechanism to help strengthen the whole family, including women within that unit. However, strengthening men on their own can significantly limit women's individual empowerment. This has implications for their personal realisation as human beings, as well as their ability to work towards a variety of other development goals. Moving forward suggests interventions which make men's empowerment *contingent* on women's empowerment. For example, this may mean supporting men as fathers and as nutrition providers (Otieno et al., 2017).

The concept of *power through* brings to the fore an under-conceptualized dimension of empowerment that highlights how empowerment of an individual is not bound to that individual only but resides also in others around her and is mediated by communities and their values. An improved understanding of the complex relational nature of empowerment can facilitate the ability of Research for Development to develop more accurate measurements of empowerment and develop effective strategies to enhance women's empowerment.

## Conflict of interest

We wish to confirm that there are no known conflicts of interest associated with this publication and there has been no significant financial support for this work that could have influenced its outcome. The financial support obtained has been mentioned in the acknowledgements. We confirm that the manuscript has been read and approved by all named authors and that there are no other persons who satisfied the criteria for authorship but are not listed. We further confirm that the order of authors listed in the manuscript has been approved by all of us. We confirm that we have given due consideration to the protection of intellectual property associated with this work and that there are no impediments to publication, including the timing of publication, with respect to intellectual property. In so doing we confirm that we have followed the regulations of our institutions concerning intellectual property. We understand that the Corresponding Author is the sole contact for the Editorial process (including Editorial Manager and direct communications with the office). She is responsible for communicating with the other authors about progress, submissions of revisions and final approval of proofs.

## Funding

This study was supported by a number of projects, programmes and institutions: the “MoreMilk: making the most of milk” project funded by the Bill and Melinda Gates Foundation and the UK Department for International Development [grant number OPP1156625]; the "MoreMilk in Tanzania" project supported by Irish Aid; the CGIAR Research Program on Agriculture for Nutrition and Health (A4NH) led by IFPRI; the CGIAR Research Program on Livestock led by ILRI - including all donors and organizations which globally support its work through their contributions to the CGIAR system (http://www.cgiar.org/about-us/our-funders/); the Participatory Plant Breeding programme at the International Centre for Agricultural Research in the Dry Areas (ICARDA).
